# Antibacterial Activity and Mechanism of Action of Aspidinol Against Multi-Drug-Resistant Methicillin-Resistant *Staphylococcus aureus*

**DOI:** 10.3389/fphar.2018.00619

**Published:** 2018-06-13

**Authors:** Xin Hua, Qin Yang, Wanjiang Zhang, Zhimin Dong, Shenye Yu, Stefan Schwarz, Siguo Liu

**Affiliations:** ^1^Division of Bacterial Diseases, State Key Laboratory of Veterinary Biotechnology, Harbin Veterinary Research Institute, Chinese Academy of Agricultural Sciences, Harbin, China; ^2^Tianjin Animal Science and Veterinary Research Institute, Tianjin, China; ^3^Institute of Microbiology and Epizootics, Department of Veterinary Medicine, Freie Universität Berlin, Berlin, Germany

**Keywords:** aspidinol, anti-MRSA activity, antimicrobial mechanism, RNA-seq, inhibit ribosomes synthesis

## Abstract

This study aimed at investigating the antibacterial activity of aspidinol, an extract from *Dryopteris fragrans* (L.) Schott, against methicillin-resistant *Staphylococcus aureus* (MRSA). MRSA isolates were treated with aspidinol to determine the differential expression of genes and associated pathways following the drug treatment. Aspidinol displayed significant anti-MRSA activity, both *in vivo* (minimum inhibitory concentration = 2 μg/mL) and *in vitro*, and achieved an antibacterial effect comparable to that of vancomycin. In the lethal septicemic mouse study, a dose of 50 mg/kg of either aspidinol or vancomycin provided significant protection from mortality. In the non-lethal septicemic mouse study, aspidinol and vancomycin produced a significant reduction in mean bacterial load in murine organs, including the spleen, lung, and liver. After treatment with aspidinol, we found through RNA-seq and RT-PCR experiments that the inhibition of the formation of ribosomes was the primary *S. aureus* cell-killing mechanism, and the inhibition of amino acid synthesis and the reduction of virulence factors might play a secondary role.

## Introduction

Methicillin-resistant *Staphylococcus aureus* (MRSA) is the most common pathogen and is associated with high morbidity and mortality in both humans and animals (VanEperen and Segreti, 2016). MRSA causes nosocomial infections, pneumonia (Woods and Colice, 2014), sepsis (Taylor, 2013), and skin infections (Mihu et al., 2015). In January 2017, MRSA was officially ranked as one of the 12 deadliest drug-resistant bacteria by WHO (2017). Antibiotics are the most effective tool to combat infections due to pathogenic bacteria. While new antimicrobial agents are becoming increasingly difficult to develop, medicine is currently unable to keep pace with the emergence of resistant bacteria (Rennie, 2012). Therefore, it is imperative to develop new agents to fight resistant bacteria, especially MRSA.

Natural products are alternative tools in the fight against drug-resistant bacteria, and synthetic approaches are unable to replace natural products (Guzman et al., 2010; Vandal et al., 2015; Farah et al., 2016). Chinese herb *Dryopteris fragrans* (L.) Schott is a deciduous perennial herbaceous plant of *Dryopteris*, it is widely distributed in many countries and is mainly grown in northeast of China (Zhong et al., 2017). As a traditional Chinese herb medicine, *D. fragrans* was used to treat a variety of diseases, especially skin diseases such as psoriasis, rashes, dermatitis, and beriberi (Huang et al., 2014). Recently, there are also reports of the antibacterial (Gao et al., 2016), anti-tumor (Zhang et al., 2012), anti-inflammatory, and antifungal activity of its extracts (Huang et al., 2014; Peng et al., 2016). Aspidinol is a phloroglucinol derivative from the stem and leaf of traditional *D. fragrans* (Zhao et al., 2014; Gao et al., 2016). To date, the research on aspidinol has been rather limited; only one publication has reported that it has anti-cancer activity (Zhao et al., 2014).

In this study, aspidinol was found to be an effective anti-MRSA agent. We have verified that aspidinol efficiently cleared intracellular bacteria and exhibited excellent *in vivo* activity, which indicated its potential for application as an antibacterial agent to treat systemic MRSA infections. Building upon this seminal work, we investigated the anti-MRSA mechanism of aspidinol. RNA-seq demonstrated that aspidinol appears to interfere with multiple biological pathways in *S. aureus* and that this interference ultimately results in the killing of MRSA.

Herein, we report for the first time the validation process of the anti-MRSA activity of aspidinol and identify its mode of action. All of the findings strongly verified the potential therapeutic application of aspidinol as a novel antibacterial agent.

## Materials and Methods

### Strains and Growth Conditions

The *S. aureus* strains ATCC 29213 and ATCC 33591 (MRSA) were obtained from the American Type Culture Collection. The clinical MSSA and MRSA isolates used in this study were provided by the First Affiliated Hospital of Harbin Medical University, Harbin, China. A complete list of bacterial strains used in this study can be found in **Supplementary Table S1**. All strains were maintained in Mueller-Hinton broth (MHB, Oxoid, Basingstoke, United Kingdom), frozen at -80°C until use, and cultured in MHB at 37°C under aerobic conditions.

### Antimicrobial Agents

Aspidinol, vancomycin, and linezolid were purchased from Sigma-Aldrich (Bornem, Belgium). Aspidinol was stored in DMSO at -20°C. All antibiotics were dissolved in ultrapure water.

### Minimum Inhibitory Concentration and Minimum Bactericidal Concentration

Broth microdilution was used to determine the minimum inhibitory concentration (MIC) according to CLSI standards M07-A9. The test medium used for most species was MHB, and the cell concentration was adjusted to approximately 5 × 10^5^ cells/mL. After 20 h of incubation at 35 ± 2°C, the MIC was read as the lowest concentration of antibiotic that inhibited visible growth of the bacteria. The minimum bactericidal concentration (MBC) was defined as the lowest concentration of aspidinol to kill *S. aureus* ATCC 33591 cells. *S. aureus* ATCC 33591 cells from the MIC assays were resuspended in fresh media and plated onto Mueller-Hinton agar (MHA). After incubating for 24 h at 37°C, the colonies were enumerated. All experiments were performed with three replicates.

### Time-Dependent Killing

An overnight culture of cells (*S. aureus* ATCC 33591) was diluted 1:5,000 in MHB and incubated at 37°C at 220 r.p.m. for 2 h after overnight culture. The bacteria cells were then treated with aspidinol, linezolid, or vancomycin at a concentration of 10 × MIC. One milliliter aliquots were removed at 2, 4, 6, 8, 12, 24, and 48 h, and 1 mL of MHB was added as a replacement. The suspension was centrifuged at 10,000 *g* for 1 min, and the pellet was resuspended in 100 mL of sterile PBS buffer. The diluted suspensions were plated onto MHA plates and incubated at 37°C overnight for CFU calculation. All experiments were performed with three replicates.

### Cytotoxicity Assay

Macrophage cells (RAW264.7) were seeded at a density of 10,000 cells per well into a 96-well cell culture plate (NEST, Nest Biotech Co. Ltd, NJ, United States) and incubated overnight at 37°C in DMEM media containing 10% fetal bovine serum (FBS). Cells were then treated with aspidinol for 24 h at different concentrations ranging from 0 to 128 μg/mL. The treated cells were washed four times with PBS, and DMEM media containing the MTS assay reagent, 3-(4,5-dimethylthiazol-2-yl)-5-(3carboxymethoxyphenyl)-2-(4-sulfophenyl)-2H-tetrazolium) (Promega, Madison, WI, United States) was then added. After 4 h of incubation at 37°C, the absorbance was measured using an ELISA microplate reader (Molecular Devices, Sunnyvale, CA, United States). The percent cell viability of the aspidinol-treated cells was calculated.

### Intracellular Infection Assay

After overnight incubation, macrophage cells (RAW 264.7) were infected with MRSA ATCC 33591 for 30 min at a multiplicity of infection (MOI) ratio of 100:1. The infected cells were then washed three times with DMEM medium containing 10 IU lysostaphin. Aspidinol (20 μg/mL), vancomycin (20 μg/mL), or linezolid (10 μg/mL) were added in complete DMEM medium containing 4 IU lysostaphin, which was then used to incubate the infected cells at 37°C (with 5% CO_2_). After 24 h, the cells were washed three times with PBS and lysed with 0.1% Triton X-100 (Sigma-Aldrich). The cell lysates were plated onto MHA plates and incubated for 24 h at 37°C, followed by CFU determination.

### Biofilm Assay

*Staphylococcus aureus* ATCC 33591 was cultured in tryptic soy broth containing 1% glucose. A biofilm formed after 24 h of incubation at 37°C. The medium was then removed, and the biofilm washed with PBS. The drugs at a concentration of 10 × MIC were added and incubated for an additional 24 h at 37°C. The 96-well plates were washed again, and the biofilms were stained with 0.1% (wt/vol) crystal violet. The 96-well plates were washed and air-dried, and finally, the biofilm mass was dissolved using 95% ethanol. A microplate reader (Bio-Tek Instruments, Inc., United States) was used to measure the intensity of crystal violet. The data were presented as the percent of biofilm mass reduction in the treated groups in relation to the control group.

### Scanning Electron Microscopy

The method of biofilm formation was carried out as described above on a glass coverslip in 24-well plates. The formed biofilm was fixed with 2.5% glutaraldehyde in 0.1 M sodium cacodylate buffer (pH 7.2) at 4°C for 10 min and then washed three times with PBS. Next, the biofilm was fixed with 1% osmic acid at room temperature for 10 min. Gradual dehydration was then carried out with ethyl alcohol (60, 70, 80, 90, 95, and 100%), and tertiary butanol was used as a displacement liquid (60, 70, 80, 90, 95, and 100%). Finally, the samples were freeze-dried overnight. The specimens were then sputter coated with gold for observation using a JSM 7500 (JEOL, Tokyo, Japan).

### Mouse Studies

Eight-week-old female BALB/c mice (Vital River, Beijing, China) were used in all of the mouse studies. The animal experiments were performed in accordance with the animal ethics guidelines and approved protocols. The animal experiment was approved by the Animal Ethics Committee of Harbin Veterinary Research Institute of the Chinese Academy of Agricultural Sciences.

### Systemic Lethal Infection

An overnight culture of *S. aureus* ATCC 33591 cells was washed and resuspended in PBS. Each mouse received an intraperitoneal (i.p.) injection (200 μL) containing the bacterial suspension (9 × 10^9^ CFU). One hour after infection, the mice were divided into seven groups (10 mice per group). The mice were intravenously injected with either aspidinol or vancomycin (5, 15, or 25 mg/kg) or the vehicle alone (10% ethanol). The treatment was provided once daily for 3 days following infection. Mortality was monitored daily for 5 days, and the moribund mice were euthanized using CO_2_ asphyxiation.

### Systemic Non-lethal Infection

The infection protocol was carried out as described above (systemic lethal infection) with the following exceptions. Each mouse received an i.p. injection containing 2 × 10^7^ CFU *S. aureus* ATCC 33591. The mice were divided into three groups (16 mice per group) and intravenously injected with either aspidinol, vancomycin (25 mg/kg), or vehicle (10% ethanol). The mice were treated once daily for 6 days. The mice were euthanized 24 h after the last administration, and the organs (including heart, lung, kidney, spleen, and liver) were excised, homogenized in MHA, and finally incubated at 37°C for 24 h for MRSA CFU counting. For evaluation of the extent of tissue damage and cellular response, histological analyses were also performed. Mice from the control group, vancomycin group, and aspidinol group that underwent the same infection protocol as the systemic non-lethal infection were submitted to histopathology examination after 6 days. The following organs were collected from each animal: heart, liver, spleen, lung, and kidneys. The tissues were fixed with 4% paraformaldehyde and then stained with hematoxylin–eosin.

### RNA-Seq Transcriptomics

*Staphylococcus aureus* ATCC 33591 was grown to an OD_600_ of 0.4 from an initial absorbance of 0.01, and aspidinol was added to a final sub-MIC concentration (1 μg/mL). Samples were collected at 1-h post treatment and preserved with RNA protect (Qiagen, United States) following the manufacturer’s instructions. Cells were pelleted by centrifugation at 5,000 *g* for 10 min at 4°C. The RNA was isolated using an RNeasy minikit (Qiagen, United States) in accordance with the manufacturer’s instructions, with the following additions. The cell pellets were homogenized in 1 mL Tris-buffered saline (TBS) (20 mM Tris, pH 7.5) containing 0.4 mg of lysostaphin and incubated at 37°C for 15 min. Subsequently, 20 mg of lysozyme in TE buffer (20 mM Tris, pH 7.5, 2 mM EDTA, pH 7.8) was added, and the sample was incubated at 25°C for 10 min. Control samples were collected from an antibiotic-free culture, and each experiment was repeated three times.

Three independently prepared RNA samples from each strain were used for RNA-Seq. Illumina sequencing was performed by Shanghai Majorbio Bio-pharm Technology Co., Ltd. (Shanghai, China) using the Illumina Hiseq2000 Truseq SBS Kit v3-HS (200 cycles) and Miseq Reagent Kit V2 (500 cycle/600 cycle) (Illumina, Inc.). The data analyses were performed using edgeR software. Genes exhibiting twofold changes in expression, which were statistically significant as determined by Student’s *t*-test (*p* < 0.05), were considered to be differentially expressed under the conditions indicated.

### Real-Time PCR

To confirm the RNA-Seq data, we selected some genes that were downregulated and assessed their relative expression levels by real-time PCR. *S. aureus* ATCC 33591 cells were cultured in the same conditions as the RNA-Seq transcriptomics experiments. qRT-PCR was performed by a two-step process. These reactions were performed using the Applied qTOWER 2.2 (Analytik Jena, Jena, Germany) Real-Time PCR System by using the following cycle parameters: 95°C for 5 min, followed by 40 cycles of 95°C for 15 s, 55°C for 15 s, and 72°C for 15 s; and one dissociation step of 95°C for 1 min, 55°C for 30 s, and 95°C for 30 s. All measurements were independently conducted three times on two separate biological isolates. All of the primers and sequences are listed in **Supplementary Table S2**.

The melting curve analysis was performed immediately after amplification to verify the specificity of the PCR amplification products. Fluorescence was measured at the end of the annealing-extension phase of each cycle. A threshold value for the fluorescence of all samples was manually set. The reaction cycle at which the PCR product exceeds this fluorescence threshold was identified as the threshold cycle (CT). The relative quantitation was calculated by the 2^-ΔΔ^CT method.

### Statistical Analysis

Statistical analyses were carried out using GraphPad Prism 5.0 (Graph Pad Software, LaJolla, CA). One-way model ANOVA was performed between the groups. In ANOVA, the observed variance is partitioned into components due to different explanatory variables. A level of *p* < 0.05 was considered to be statistically significant.

## Results

### Antibacterial Activity of Aspidinol

The antimicrobial activity of aspidinol was tested against clinical isolates of *S. aureus*, including drug-resistant strains, the MICs and MBCs of which are given in **Table 1**. Aspidinol showed potent bactericidal activity against MSSA and MRSA, with MICs and MBCs ranging from 0.25 to 2 μg/mL and 0.5 to 4 μg/mL, respectively.

**Table 1:** MIC and MBC of aspidinol against *Staphylococcus* strains.

Strains	Aspidinol	Oxacillin	Aspidinol	Oxacillin
		
	MIC (μg/mL)		MBC (μg/mL)	
MSSA standard strain ATCC 29213	0.5	0.25	1	0.5
MSSA clinical isolates (20 isolates)	0.25–2	0.25–1	0.25–4	0.5–2
MRSA standard strain ATCC 33591	2	128	4	>128
MRSA clinical isolates (19 isolates)	0.5–2	32 to >128	1–8	64 to >128

**FIGURE 1:**
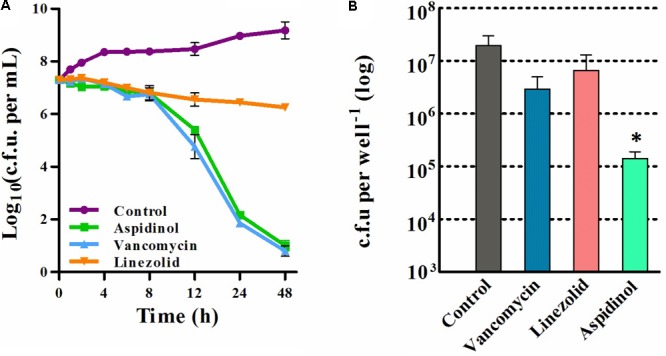
Time-kill analysis and the intracellular bacterial killing activity of aspidinol. **(A)** Time-kill kinetics of aspidinol against *S. aureus* ATCC 33591. The error bars show the standard deviations of the results from three independent biological repeats. **(B)** *S. aureus* ATCC 33591-infected RAW264.7 cells were treated with aspidinol and control antibiotics (vancomycin or linezolid) for 24 h, and the percent bacterial reduction was calculated compared to that of the untreated control groups. The results are given as the mean ± SD (*n* = 3). Two-tailed Student’s *t*-test was employed, and ^∗^*p*-values of ≤0.05 are considered to be significant.

After confirming that aspidinol possessed excellent antimicrobial activity against *S. aureus*, we next assessed the killing kinetics of aspidinol. As depicted in **Figure 1A**, aspidinol also showed excellent bactericidal activity against *S. aureus*.

### Cytotoxicity of Aspidinol

Cytotoxicity tests indicated that the toxicity of aspidinol toward macrophage cells (RAW264.7) was negligible. At 128 μg/mL, i.e., the highest dose tested, aspidinol showed no toxicity to RAW264.7 cells. The dose response curve of aspidinol in the cytotoxicity assay can be found in **Supplementary Figure S2**.

### Aspidinol Efficiently Clears Intracellular Bacteria

Eliminating intercellular *S. aureus* has long been considered to be key to clinical success because *S. aureus* is capable of entering multiple mammalian cells, thereby evading the antibiotic therapy. The ability to internalize into mammalian cells might result in the long-term colonization of the host and explain clinical failures. Lehar et al. have confirmed this hypothesis, showing that three major antibiotics commonly used for clinical standard care (vancomycin, daptomycin, and linezolid) failed to kill the highly virulent community-acquired *S. aureus* ATCC 33591 strain (Lehar et al., 2015).

As aspidinol exhibited potent anti-MRSA activity, we explored the ability of aspidinol to permeate cellular membranes and kill MRSA located inside eukaryotic cells. To investigate the efficacy of aspidinol in eliminating intracellular MRSA, macrophage cells (RAW264.7) infected with *S. aureus* ATCC 33591 were employed. As depicted in **Figure 1B**, intracellular *S. aureus* ATCC 33591 exposed to aspidinol (20 μg/mL) were efficiently killed, and the intracellular bacteria decreased by 100-fold. In contrast, conventional antibiotics such as linezolid (20 μg/mL) and vancomycin (10 μg/mL) were shown to reduce the bacterial burden inside the infected macrophages by 5- to 10-fold. From the above data, aspidinol was confirmed to be capable of eradicating MRSA infection in macrophage cells.

### Inhibitory Effect of Aspidinol on MRSA Biofilm Formation

**FIGURE 2:**
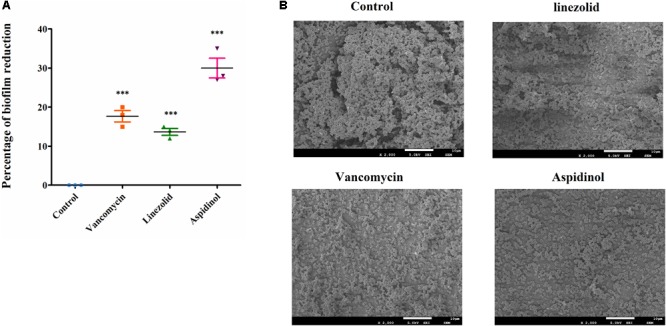
Anti-biofilm activity of aspidinol. **(A)** The effects of aspidinol and antibiotics (linezolid and vancomycin) on established biofilms of *S. aureus* ATCC 33591 were evaluated. The pre-formed biofilms were treated with linezolid, vancomycin, or aspidinol and then stained with crystal violet. The optical density of the dissolved crystal violet was measured using a spectrophotometer. The values are the mean of the triplicate samples with standard deviation bars. The results are given as the mean ± SD (*n* = 3). Two-tailed Student’s *t*-test was employed, and ^∗∗∗^ means *p*-values ≤ 0.005. **(B)** Scanning electron microscopy images showing the structure of MRSA biofilms treated with aspidinol and antibiotics at 24 h. Magnifications, ×2000.

The biofilms were stained with crystal violet after treatment with aspidinol, and the absorbance was measured at 570 nm for mass estimation. Compared to linezolid and vancomycin, aspidinol was not able to significantly reduce adherent biofilms of *S. aureus* ATCC 33591. At a concentration of 256 μg/mL, which corresponds to the 128-fold MIC, aspidinol reduced the biofilm mass by approximately by 30%. The control antibiotics, linezolid (256 μg/mL, i.e., 128-fold MIC) and vancomycin (128 μg/mL, i.e., 128-fold MIC), were able to reduce the biofilm mass by only 20% (**Figure 2A**).

The morphology of *S. aureus* ATCC 33591 biofilms on the surface of coverslips was observed using scanning electron microscope (SEM). Under a 2,000-fold magnification, the biofilm was shown to be composed of many multilayered MRSA colonies. The SEM analysis results were consistent with those of the crystal violet staining observations. At a concentration of 256 μg/mL of aspidinol, linezolid, and vancomycin, we observed a decrease in density of the MRSA biofilms (**Figure 2B**).

### *In Vivo* Efficacy of Aspidinol

**FIGURE 3:**
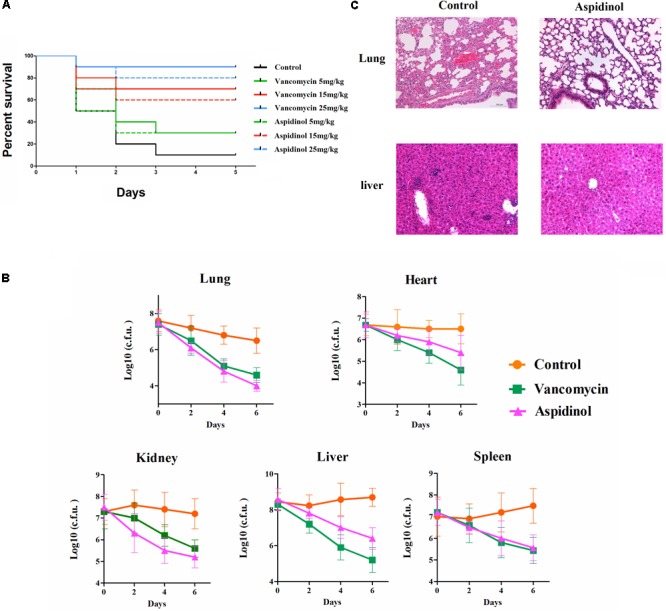
Aspidinol is effective in a mouse model of MRSA septicemic infection. **(A)** Ten mice per group were infected (i.p.) with a lethal dose of *S. aureus* ATCC 33591 and intravenously injected with aspidinol, vancomycin (5, 15, or 25 mg/kg), or the vehicle alone for 5 days (one dose per day). Mice were monitored for 5 days and the percentage survival was calculated. The statistical significance was calculated in order to compare treated to control groups. **(B)** Six mice per group were infected (i.p.) with a non-lethal dose of *S. aureus* ATCC 33591 and intravenously injected with aspidinol, vancomycin (25 mg/kg) or the vehicle alone for 6 days (one dose per day). Twenty-four hours after the last treatment, the mice were euthanized, and their organs were excised and homogenized in TSB to count viable MRSA colonies. The number of CFUs from each mouse is plotted as individual points. Values are the mean of triplicate samples with standard deviation bars. **(C)** Histological evaluation of lung and liver of mice infected with *S. aureus* ATCC 33591 receiving no treatment or a treatment with aspidinol. Both lung and liver in the control group demonstrated acute inflammation; no apparent pathological changes were observed in the treatment group.

To determine the role of aspidinol against MRSA *in vivo*, both a lethal and a non-lethal systemic MRSA infection model were used. In the lethal systemic study, mice were infected intraperitoneally with MRSA ATCC 33591 (2.95 × 10^9^ CFU per mouse). Both aspidinol and vancomycin provided significant protection from mortality (**Figure 3A**). The survival rate of infected mice varied in a concentration-dependent manner, with the survival rate improving dramatically when the dose of aspidinol increased. Approximately 80% of mice that received a higher dose of aspidinol (25 mg/kg) survived for 5 days. These results exhibited the *in vivo* activity of aspidinol in protecting mice from septicemic MRSA infection.

Next, the efficacy of aspidinol against MRSA ATCC 33591 in a non-lethal model was investigated. In brief, mice were first infected with a non-lethal dose of MRSA ATCC 33951 (4.20 × 10^7^ CFU/mouse), and then each group of mice received two oral doses of aspidinol, vancomycin (25 mg/kg), or the vehicle alone. As depicted in **Figure 3B**, aspidinol and vancomycin produced a significant reduction in mean bacterial load in different organs. In particular, both treatments reduced the mean bacterial load by more than 1000-fold in the lung. The histopathological inspection 6 days after infection with the non-lethal dose of MRSA ATCC 33951 revealed no changes in the heart, spleen, and kidney. While the animals showed moderate histopathological alterations in lung and liver in the control group, after treatment with aspidinol or vancomycin, there were no obvious histopathological alterations (**Figure 3C**).

### RNA-Seq

To better understand the mechanism of the anti-MRSA effect of aspidinol, the underlying differential expression of MRSA genes was analyzed after treatment with aspidinol.

**FIGURE 4:**
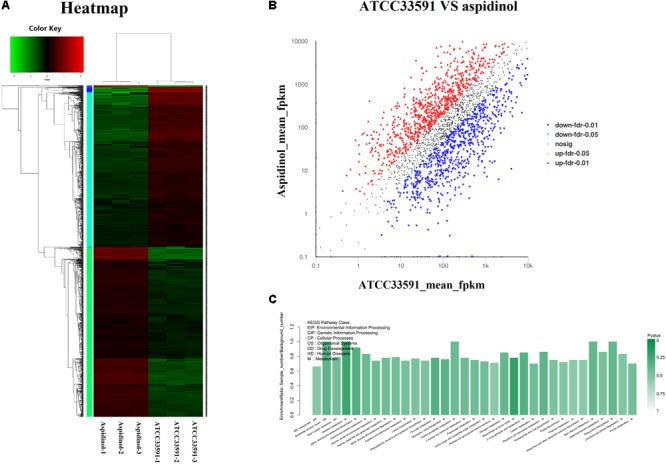
RNA-Seq gene expression results for *S. aureus* ATCC 33591 cells treated and not treated with aspidinol. **(A)** A heatmap generated comparing aspidinol-treated cells to untreated control *S. aureus* ATCC 33591 cells is shown. Triplicate samples were used for each group. **(B)** Volcano plot analyses of unigenes in aspidinol and the ATCC 33591 group. **(C)** Differentially expressed genes enriched in the KEGG pathway.

A heatmap and volcano plot analyses revealed the differential gene expression for both untreated and treated MRSA (**Figures 4A,B**). A total of 2952 genes were identified; among these, 1147 genes were upregulated and 1183 were downregulated during aspidinol treatment. After quantile normalization of the FPKM values followed by Student’s *t*-test at *p* = 0.05 and the selection of DEGs with at least a twofold change in their expression in response to aspidinol treatment, we identified 949 DEGs (456 upregulated and 493 downregulated) with highly significant expression patterns before and after the treatment. All the sequencing reads have been submitted to the NCBI short-read archive (SRA) with accession number SRP129492.

#### KEGG Pathway Analysis of the DEGs

KEGG pathway analysis was used to further clarify the biological function of aspidinol. Overall, DEGs were significantly enriched in 55 KEGG pathways, meeting the criteria of *p*-values <0.05 (**Figure 4C**, more clearer pictures can be found in **Supplementary Figure S1**). The KEGG pathways that showed the highest levels of significance were the lysine biosynthesis pathway (ko00300), the valine, leucine, and isoleucine biosynthesis pathways (ko00290), nitrogen metabolism (ko00910), galactose metabolism (ko00052), carbon metabolism (ko01200), and amino sugar and nucleotide sugar metabolism (ko00520), suggesting that these pathways may play important roles in the antibacterial processes of aspidinol.

#### Genes Involved in the Synthesis of Amino Acid

The analysis of genes downregulated by aspidinol in *S. aureus* ATCC 33591 showed a significant decrease in the expression of the genes involved in the synthesis of amino acids. The synthesis of amino acids is important for the survival of bacteria. Related genes that act in the synthesis of valine, leucine, isoleucine, and lysine were downregulated after treatment with aspidinol. Genes encoding important intermediates in amino acid synthesis, such as *ilvC* and *ilvD*, were also downregulated 3.47- and 4.73-fold, respectively. In addition, the leucine synthesis genes *leuABCD* were also downregulated 2.6-, 3.08-, 2.58-, and 2.49-fold, respectively. The gene *lysC*, which encodes an aspartate kinase, was downregulated 5.77-fold. In addition to amino acid biosynthesis, genes involved in peptide import were also significantly downregulated. The genes *oppABCDF*, encoding a peptide transport system, were downregulated 1.15-, 7.37-, 3.68-, 2.30-, and 1.40-fold, respectively.

#### Genes Involved in Ribosome Synthesis

Four genes involved in structural components of ribosomes, 30S ribosomal protein S20 *rpsT* (-4.01-fold), 50S ribosomal protein L1 *rplA* (-4.34-fold), L20 *rplT* (-4.11-fold), and L32 (-2.33-fold), were downregulated after treatment with aspidinol. Nevertheless, three genes involved in structural components of ribosomes, 30S ribosomal protein S11 *rpsK* (2.3-fold), 50S ribosomal protein L17 *rplQ* (2.33-fold), and L7/L12 *SAV1268* (2.22-fold) were upregulated. In addition, some genes involved in ribosome synthesis were significantly changed. The gene expression of the ribosomal large subunit pseudouridine synthase D *rluD*, ribosomal-protein-serine N-acetyltransferase *rimL*, and ribosome-binding factor A *rbfA* decreased by 4.66-, 2.82-, and 2.9-fold, respectively. The ribosomal subunit interface protein *SAV0752* and ribosomal large subunit pseudouridine synthase B *SAV1493* were increased by 4.21- and 2.32-fold, respectively.

#### Repression of Genes Involved in Iron Transport and Key Virulence Factors

Aspidinol also significantly repressed genes involved in iron ABC transporter synthesis. The constituents of the ferrichrome ABC transporter system comprising *fhuABG* were downregulated by aspidinol 4.93-, 4.12-, and 3.87-fold, respectively. The genes encoding metal iron ABC transport *mtsABC* were downregulated 4.06-, 5.31-, and 8.59-fold, respectively. Heme transporter *IsdA* was downregulated 4.34-fold. After treatment with aspidinol, the repression of key virulence factors, exfoliative toxins *eta* and IgG-binding protein *sbi*, were downregulated 4.82- and 2.82-fold, respectively.

#### Genes Involved in the Metabolism of Energy

Aspidinol-treated *S. aureus* ATCC 700699 showed an increase in the expression of genes involved in basic metabolic processes, including metabolism of nitrogen, galactose, and carbon. The genes involved in nitrogen metabolism, including *nirBD, narGHJ*, and *arc*, were upregulated 5.36-, 4.68-, 4.68-, 3.81-, and 9.91-fold, respectively. The *lacABCDEFG* genes encoding a galactose-6-phosphate isomerase were upregulated 5.68-, 4.28-, 4.22-, 5.72-, 3.6-, 6.22-, and 5.54-fold, respectively. In addition, the carbon metabolism genes *sdaA, sdaB, lpd, prsA*, and *tdcB* were also upregulated 4.29-, 3.67-, 2.84-, 2.75-, and 5.1-fold, respectively.

#### Genes Involved in β-Lactam Resistance

Most genes related to the β-lactam resistance pathway were significantly downregulated after treatment with aspidinol, including beta-lactamase repressor *blaI*, beta-lactamase regulatory gene *blaR1*, and methicillin resistance regulatory genes *mecI* and *mecR1*, which are key genes attributed to drug resistance of β-lactam antibiotics. After treatment, *blaR1, blaI, mecR1, mecI* were downregulated 5.6-, 4.49-, 3.06-, and 4.35-fold, respectively, relative to their expression in the untreated control.

### RNA-Seq Results Were Verified by qRT-PCR

**FIGURE 5:**
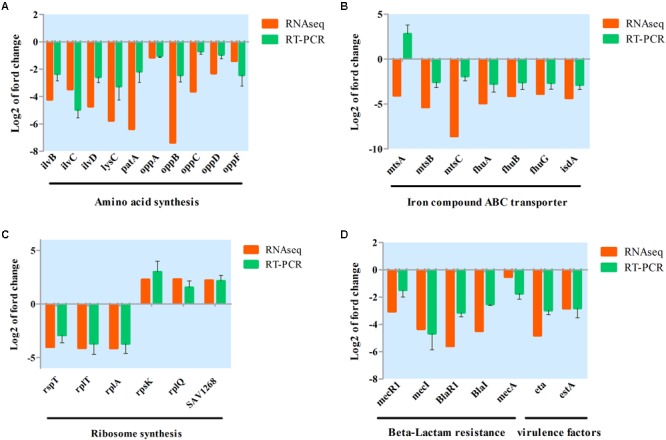
Validation of RNA-seq data for selected genes by real-time PCR. **(A)** Differentially expressed genes involved in amino acid synthesis; **(B)** differentially expressed genes involved in iron ABC transporter synthesis; **(C)** differentially expressed genes involved in ribosome synthesis; **(D)** differentially expressed genes involved in beta-lactam resistance and virulence factor.

A qRT-PCR assay was conducted to further validate transcriptional changes induced by aspidinol and observed in the RNA-seq experiments. After treatment with sub-MIC (1 μg/mL) of aspidinol, 30 different genes were chosen, including *oppABCDF, fhuABG*, and *ilvBCD* among others (**Figure 5**), to determine changes in their transcript accumulation. There was no obvious variation between qRT-PCR and transcriptomic data in the expression patterns except for *mtsA*. Although the exact fold change difference in expression for each gene by qRT-PCR was different from the RNA-seq data, similar trends were observed, which suggests that there is a relatively high consistency between the RNA-seq and qRT-PCR results.

## Discussion

*Staphylococcus aureus*, in particular MRSA, has acquired numerous antimicrobial resistance genes, rendering these isolates multi-resistant. This development of resistance and multi-resistance is likely to have the effect that the currently available antibiotics will not be as efficacious in the near future (Rodvold and McConeghy, 2014; Shenoy et al., 2014; Stahlmann, 2014). Unfortunately, the development of novel antibiotics and their introduction into clinical use cannot keep pace with the emergence of resistant pathogens. Relatedly, pharmaceutical companies have increasingly withdrawn investment in the development of new antimicrobial agents. Despite this situation and the associated challenges to healthcare, new strategies, and alternative methods for anti-infective therapy need to be explored.

In preliminary experiments, aspidinol was chosen as a study object through antibacterial activity screening. Aspidinol exhibited an excellent anti-MRSA activity *in vitro* and no cytotoxicity. Time-kill assays revealed that the anti-MRSA effect of aspidinol was nearly identical to that seen with vancomycin and linezolid. In previous reports of Mu, two analogs of aspidinol, aspidinol C and D, were reported to have significant anti-MRSA activity. The MICs of aspidinol C and D against MRSA were 2 and 4 μg/mL, respectively (The structures of aspidinol, aspidinol C and D can be found in **Supplementary Figure S3**). However, there was no further in-depth study of aspidinol C and D except for determining their MIC value (Mu et al., 2012). Therefore, it could be speculated that aspidinol may also have excellent anti-MRSA activity due to it similar chemical structure. Indeed, compared with aspirinol C and D, aspirinol shows stronger anti-MRSA activity.

Once MRSA internalizes into eukaryotic cells, intracellular persistence will prolong the presence of the pathogen in the host and result in a weakening of the host defenses (Lemaire et al., 2008). Because of their inability to penetrate the cell membranes, most antibiotics, including vancomycin, cannot effectively combat intracellular MRSA (Garzoni and Kelley, 2009; Seleem et al., 2009; Tenover and Goering, 2009; Thangamani et al., 2015). Vancomycin has been reported to have a failure rate of greater than 40% in treating *S. aureus* pneumonia (Rubinstein et al., 2008). In this study, aspidinol exhibited excellent anti-MRSA activity inside macrophages; this activity was 100 times better than that of vancomycin. However, compared to vancomycin and linezolid, aspidinol did not significantly reduce adherent MRSA biofilms. This outcome suggests that aspidinol may be more suitable for systemic MRSA infectious diseases, such as septicemia, rather than skin infections.

The *in vivo* efficacy of aspidinol was assessed in two murine MRSA systemic infection models (non-lethal and lethal). Both the non-lethal and lethal mouse studies confirmed that aspidinol retains its antibacterial activity *in vivo*. The results were consistent with the results of the anti-MRSA activity *in vitro*, proving that aspidinol was able to effectively prevent and treat systemic infections caused by MRSA.

To better understand the pathway of the anti-MRSA effect of aspidinol, RNA-seq experiments were conducted. The analysis of transcriptomic changes caused by aspidinol treatment showed that the expression of a number of genes involved in ribosome synthesis was up- and downregulated upon treatment with aspidinol. The results indicated that ribosome synthesis was more likely to be inhibited after treatment with aspidinol and result in the inhibition of protein synthesis in bacteria. The increase in the expression of genes involved in ribosome synthesis may therefore be in response to stress. The inhibition of the formation of the ribosome, which is composed of the 30S and 50S subunits, is an important antibacterial mechanism, such as in linezolid (Colca et al., 2003; Ippolito et al., 2008; Wilson et al., 2008). Thus, the changes of genes involved in ribosome synthesis may contribute to the antimicrobial activity of aspidinol.

Iron transport was also found to be suppressed. With the possible exception of lactobacilli, all bacteria require iron for growth because it plays a vital role in the biological processes of bacteria (Sebulsky et al., 2000). MtsABC proteins were reported to be associated with metal binding and transmembrane transport. A metal solute-binding lipoprotein and an ATP-binding protein were encoded by *mtsA* and *mtsB*, respectively, while *mtsC* encodes a transmembrane permease protein (Sun et al., 2008, 2009; Zou et al., 2010). Thus, the *mtsABC* operon is also essential for the survival of bacteria (Janulczyk et al., 2003); *mtsA*, as a metal ion transporter, is responsible for iron release and transmembrane uptake and interacts with membrane components. The Fhu system is the best-characterized system in *S. aureus* for ferric hydroxamate uptake and is composed of up to five genes (Sebulsky et al., 2000). The genes *fhuC, fhuB*, and *fhuG* reside in an operon that encodes an ATP-binding cassette (ABC) transporter; *fhuC* encodes an ATPase at the inner side of the cytoplasmic membrane, and *fhuB* and *fhuG* are membrane-spanning proteins (Pramanik and Braun, 2006; Speziali et al., 2006). When treated with aspidinol, *mtsABC* and *fhuABCDF* are downregulated, resulting in decreased iron transport and further reducing the energy supply of ribosome synthesis, which also might contribute to MRSA killing.

Moreover, amino acid biosynthesis was downregulated after aspidinol treatment, including the leucine, isoleucine, and valine synthesis. Pathway analysis using the transcriptome data showed that the expression of the valine, leucine, and isoleucine biosynthesis system was decreased. The downregulated *ilv* and *leu* operons have been reported to encode leucine, isoleucine, and valine intermediates in the synthesis of these amino acids in *E. coli* (Miyanoiri et al., 2016). According to our results, we speculate that these operons may play the same role, although there are no reports regarding their function in *S. aureus*. Components of other amino acid pathways, such as *lysC*, were apparently downregulated by aspidinol; *lysC* is the primary gene for lysine biosynthesis and is involved in the biosynthesis of lysine from aspartic acid (Wiltshire and Foster, 2001; Nishi et al., 2004). In addition to amino acid biosynthesis, the *oppABCDF* genes were also downregulated in response to aspidinol. The Opp system is essential for the uptake of nutrients by bacteria (Gardan et al., 2009) and has been shown to supply bacteria with exogenous peptides that serve as an amino acid resource (Zhao et al., 2016). In this study, downregulation of amino acid biosynthesis pathways after treatment with aspidinol could be attributed to the killing of MRSA.

Aspidinol also significantly repressed genes involved in the virulence of MRSA. Following aspidinol treatment at sub-inhibitory concentrations, exfoliative toxin *eta* and IgG-binding protein *sbi* were measurably downregulated. Our data suggest that in addition to killing ATCC 33591, it is very likely that aspidinol reduced the virulence of the infecting bacterial cells as a secondary effect.

According to the data analysis, genes associated with β-lactam resistance were significantly reduced after treatment with aspidinol. Through the analysis of the transcription data, we found that the expression levels of genes associated with β-lactam resistance (*mecI, mecR1, blaZ*, and *blaR1*) were significantly reduced after treatment with aspidinol; *mecA* coding for PBP2a (a low affinity penicillin binding protein) is responsible for methicillin resistance in *S. aureus* and other staphylococci (Wilke et al., 2004; Staude et al., 2015). While transcription of *mecA* is regulated by *mecI* and *mecR1* (Wilke et al., 2004; Aedo and Tomasz, 2016), *blaR1* and *blaI* are involved in the regulation of *blaZ* (Thumanu et al., 2006). Based on our current understanding, blocking of *blaR1* and *mecR1* is helpful to restore the susceptibility of MRSA to β-lactam antibiotics. Hou et al. (2011) have designed specific anti-mecR1 and anti-blaR1 deoxyribozymes and confirmed their function in restoring the β-lactam susceptibility of MRSA.

According to the transcriptome data, the expression of *mecA* was decreased 0.52-fold, but there were no statistically significant differences. However, the RT-PCR results showed that *mecA* was downregulated 1.75-fold after treatment with aspidinol. Subsequently, a combination administration experiment was conducted to test whether a sub-MIC concentration of aspidinol was able to restore the susceptibility to β-lactams. In this experiment, oxacillin was co-administered with 1 μg/mL aspidinol, and the oxacillin MIC decreased from 256 to 0.5 μg/mL. This observation might provide a first hint toward the use of aspidinol as a combination drug to restore the β-lactam susceptibility. We theorize that aspidinol is able to inhibit the expression of *mecA*-related genes to restore susceptibility to β-lactam antibiotics.

Genes involved in some metabolic pathways, such as carbon metabolism and nitrogen metabolism, were upregulated after treatment with aspidinol, which could be attributed to metabolic disorders of bacteria in the process of dying.

Although the validation of the effects of aspidinol on metabolic pathways has been carried out by RT-PCR, further studies are still needed to more thoroughly understand the antibacterial mechanisms of aspidinol.

However, these data were not sufficient to fully elucidate the antibacterial mechanisms of aspidinol against MRSA. In future studies, we will focus on the identification of potential drug targets to clearly reveal the mechanism of action.

## Conclusion

In this study, the anti-MRSA activity of aspidinol, both *in vitro* and *in vivo*, was reported for the first time. The mechanism of aspidinol’s anti-MRSA effect was also investigated. In particular, RNA-seq was applied for the analysis of differentially expressed genes after treatment with aspidinol. When combined with verification experiments, the results suggest that the inhibition of the formation of ribosomes was the primary *S. aureus* cell-killing mechanism, and the inhibition of amino acid synthesis and the reduction of virulence factors were considered to be a secondary effect.

## Author Contributions

XH and SL responsible for experimental design. XH, QY, and ZD responsible for experimental operation. XH, WZ, and SY responsible for writing the manuscript. SS responsible for reviewing the manuscript.

## Conflict of Interest Statement

The authors declare that the research was conducted in the absence of any commercial or financial relationships that could be construed as a potential conflict of interest.

## Supplementary Material

FIGURE S1 Differentially expressed genes enriched in the KEGG pathway for *S. aureus* ATCC 33591 cells treated and not treated with aspidinol.

FIGURE S2 Cell survival curve after treated with aspidinol.

FIGURE S3 Chemical structure of aspidinol, aspidinol C and aspidinol D.

TABLE S1 Strain list.

TABLE S2 Primers used in this study.

## References

[B1] Aedo S. Tomasz A. (2016). Role of the stringent stress response in the antibiotic resistance phenotype of methicillin-resistant *Staphylococcus aureus*. *Antimicrob. Agents Chemother.* 60 2311–2317. 10.1128/AAC.02697-15 26833147 PMC4808156

[B2] Colca J. R. Mcdonald W. G. Waldon D. J. Thomasco L. M. Gadwood R. C. Lund E. T. (2003). Cross-linking in the living cell locates the site of action of oxazolidinone antibiotics. *J. Biol. Chem.* 278 21972–21979. 10.1074/jbc.M302109200 12690106

[B3] Farah S. I. Abdelrahman A. North E. J. Chauhan H. (2016). Opportunities and challenges for natural products as novel antituberculosis agents. *Assay Drug Dev. Technol.* 14 29–38. 10.1089/adt.2015.673 26565779

[B4] Gao C. Guo N. Li N. Peng X. Wang P. Wang W. (2016). Investigation of antibacterial activity of aspidin BB against *Propionibacterium acnes*. *Arch. Dermatol. Res.* 308 79–86. 10.1007/s00403-015-1603-x 26596576

[B5] Gardan R. Besset C. Guillot A. Gitton C. Monnet V. (2009). The oligopeptide transport system is essential for the development of natural competence in *Streptococcus thermophilus* strain LMD-9. *J. Bacteriol.* 191 4647–4655. 10.1128/JB.00257-09 19447907 PMC2704715

[B6] Garzoni C. Kelley W. L. (2009). *Staphylococcus aureus*: new evidence for intracellular persistence. *Trends Microbiol.* 17 59–65. 10.1016/j.tim.2008.11.005 19208480

[B7] Guzman J. D. Gupta A. Evangelopoulos D. Basavannacharya C. Pabon L. C. Plazas E. A. (2010). Anti-tubercular screening of natural products from Colombian plants: 3-methoxynordomesticine, an inhibitor of MurE ligase of *Mycobacterium tuberculosis*. *J. Antimicrob. Chemother.* 65 2101–2107. 10.1093/jac/dkq313 20719764

[B8] Hou Z. Zhou Y. Wang H. Bai H. Meng J. Xue X. (2011). Co-blockade of mecR1/blaR1 signal pathway to restore antibiotic susceptibility in clinical isolates of methicillin-resistant *Staphylococcus aureus*. *Arch. Med. Sci.* 7 414–422. 10.5114/aoms.2011.23404 22295022 PMC3258742

[B9] Huang Y. H. Zeng W. M. Li G. Y. Liu G. Q. Zhao D. D. Wang J. (2014). Characterization of a new sesquiterpene and antifungal activities of chemical constituents from *Dryopteris fragrans* (L.) Schott. *Molecules* 19 507–513. 10.3390/molecules19010507 24451246 PMC6272047

[B10] Ippolito J. A. Kanyo Z. F. Wang D. Franceschi F. J. Moore P. B. Steitz T. A. (2008). Crystal structure of the oxazolidinone antibiotic linezolid bound to the 50S ribosomal subunit. *J. Med. Chem.* 51 3353–3356. 10.1021/jm800379d 18494460

[B11] Janulczyk R. Ricci S. Bjorck L. (2003). MtsABC is important for manganese and iron transport, oxidative stress resistance, and virulence of *Streptococcus pyogenes*. *Infect. Immun.* 71 2656–2664. 10.1128/IAI.71.5.2656-2664.2003 12704140 PMC153223

[B12] Lehar S. M. Pillow T. Xu M. Staben L. Kajihara K. K. Vandlen R. (2015). Novel antibody-antibiotic conjugate eliminates intracellular *S. aureus*. *Nature* 527 323–328. 10.1038/nature16057 26536114

[B13] Lemaire S. Olivier A. Van Bambeke F. Tulkens P. M. Appelbaum P. C. Glupczynski Y. (2008). Restoration of susceptibility of intracellular methicillin-resistant *Staphylococcus aureus* to beta-lactams: comparison of strains, cells, and antibiotics. *Antimicrob. Agents Chemother.* 52 2797–2805. 10.1128/AAC.00123-08 18519727 PMC2493141

[B14] Mihu M. R. Roman-Sosa J. Varshney A. K. Eugenin E. A. Shah B. P. Lee H. H. (2015). Methamphetamine alters the antimicrobial efficacy of phagocytic cells during methicillin-resistant *Staphylococcus aureus* skin infection. *MBio* 6:e1622-15. 10.1128/mBio.01622-15 26507236 PMC4626859

[B15] Miyanoiri Y. Ishida Y. Takeda M. Terauchi T. Inouye M. Kainosho M. (2016). Highly efficient residue-selective labeling with isotope-labeled Ile, Leu, and Val using a new auxotrophic *E. coli* strain. *J. Biomol. NMR* 65 109–119. 10.1007/s10858-016-0042-0 27272978

[B16] Mu Q. Zeng C. H. Gibbons S. Osman C. (2012). Aspidin Phenolic Compounds and Their Use in the Orparation of Anti-drug Resistant. CN Patent No. 102464578 A. Peking: State Intellectual Property Office of the People’s Republic of China.

[B17] Nishi H. Komatsuzawa H. Fujiwara T. Mccallum N. Sugai M. (2004). Reduced content of lysyl-phosphatidylglycerol in the cytoplasmic membrane affects susceptibility to moenomycin, as well as vancomycin, gentamicin, and antimicrobial peptides, in *Staphylococcus aureus*. *Antimicrob. Agents Chemother.* 48 4800–4807. 10.1128/AAC.48.12.4800-4807.2004 15561859 PMC529239

[B18] Peng B. Bai R. F. Li P. Han X. Y. Wang H. Zhu C. C. (2016). Two new glycosides from *Dryopteris fragrans* with anti-inflammatory activities. *J. Asian Nat. Prod. Res.* 18 59–64. 10.1080/10286020.2015.1121853 26700189

[B19] Pramanik A. Braun V. (2006). Albomycin uptake via a ferric hydroxamate transport system of *Streptococcus pneumoniae* R6. *J. Bacteriol.* 188 3878–3886. 10.1128/JB.00205-06 16707680 PMC1482914

[B20] Rennie R. P. (2012). Current and future challenges in the development of antimicrobial agents. *Handb. Exp. Pharmacol.* 211 45–65. 10.1007/978-3-642-28951-4_4 23090595

[B21] Rodvold K. A. McConeghy K. W. (2014). Methicillin-resistant *Staphylococcus aureus* therapy: past, present, and future. *Clin. Infect. Dis.* 58(Suppl. 1) S20–S27. 10.1093/cid/cit614 24343828

[B22] Rubinstein E. Kollef M. H. Nathwani D. (2008). Pneumonia caused by methicillin-resistant *Staphylococcus aureus*. *Clin. Infect. Dis.* 46 S378–S385. 10.1086/533594 18462093

[B23] Sebulsky M. T. Hohnstein D. Hunter M. D. Heinrichs D. E. (2000). Identification and characterization of a membrane permease involved in iron-hydroxamate transport in *Staphylococcus aureus*. *J. Bacteriol.* 182 4394–4400. 10.1128/JB.182.16.4394-4400.2000 10913070 PMC94608

[B24] Seleem M. N. Jain N. Pothayee N. Ranjan A. Riffle J. S. Sriranganathan N. (2009). Targeting *Brucella melitensis* with polymeric nanoparticles containing streptomycin and doxycycline. *FEMS Microbiol. Lett.* 294 24–31. 10.1111/j.1574-6968.2009.01530.x 19493005

[B25] Shenoy E. S. Paras M. L. Noubary F. Walensky R. P. Hooper D. C. (2014). Natural history of colonization with methicillin-resistant *Staphylococcus aureus* (MRSA) and vancomycin-resistant *Enterococcus* (VRE): a systematic review. *BMC Infect. Dis.* 14:177. 10.1186/1471-2334-14-177 24678646 PMC4230428

[B26] Speziali C. D. Dale S. E. Henderson J. A. Vines E. D. Heinrichs D. E. (2006). Requirement of *Staphylococcus aureus* ATP-binding cassette-ATPase FhuC for iron-restricted growth and evidence that it functions with more than one iron transporter. *J. Bacteriol.* 188 2048–2055. 10.1128/JB.188.6.2048-2055.2006 16513734 PMC1428144

[B27] Stahlmann R. (2014). Antibiotics for treatment of infections by methicillin-resistant *Staphylococcus aureus* (MRSA). *Pneumologie* 68 676–684. 10.1055/s-0034-1377747 25290922

[B28] Staude M. W. Frederick T. E. Natarajan S. V. Wilson B. D. Tanner C. E. Ruggiero S. T. (2015). Investigation of signal transduction routes within the sensor/transducer protein BlaR1 of *Staphylococcus aureus*. *Biochemistry* 54 1600–1610. 10.1021/bi501463k 25658195 PMC4691190

[B29] Sun X. Baker H. M. Ge R. Sun H. He Q. Y. Baker E. N. (2009). Crystal structure and metal binding properties of the lipoprotein MtsA, responsible for iron transport in *Streptococcus pyogenes*. *Biochemistry* 48 6184–6190. 10.1021/bi900552c 19463017

[B30] Sun X. Ge R. Chiu J. F. Sun H. He Q. Y. (2008). Lipoprotein MtsA of MtsABC in *Streptococcus pyogenes* primarily binds ferrous ion with bicarbonate as a synergistic anion. *FEBS Lett.* 582 1351–1354. 10.1016/j.febslet.2008.03.020 18364240

[B31] Taylor A. R. (2013). Methicillin-resistant *Staphylococcus aureus* infections. *Prim. Care* 40 637–654. 10.1016/j.pop.2013.06.002 23958361

[B32] Tenover F. C. Goering R. V. (2009). Methicillin-resistant *Staphylococcus aureus* strain USA300: origin and epidemiology. *J. Antimicrob. Chemother.* 64 441–446. 10.1093/jac/dkp241 19608582

[B33] Thangamani S. Younis W. Seleem M. N. (2015). Repurposing clinical molecule ebselen to combat drug resistant pathogens. *PLoS One* 10:e0133877. 10.1371/journal.pone.0133877 26222252 PMC4519285

[B34] Thumanu K. Cha J. Fisher J. F. Perrins R. Mobashery S. Wharton C. (2006). Discrete steps in sensing of beta-lactam antibiotics by the BlaR1 protein of the methicillin-resistant *Staphylococcus aureus* bacterium. *Proc. Natl. Acad. Sci. U.S.A.* 103 10630–10635. 10.1073/pnas.0601971103 16815972 PMC1502283

[B35] Vandal J. Abou-Zaid M. M. Ferroni G. Leduc L. G. (2015). Antimicrobial activity of natural products from the flora of Northern Ontario, Canada. *Pharm. Biol.* 53 800–806. 10.3109/13880209.2014.942867 25697605

[B36] VanEperen A. S. Segreti J. (2016). Empirical therapy in methicillin-resistant *Staphylococcus aureus* infections: an up-to-date approach. *J. Infect. Chemother.* 22 351–359. 10.1016/j.jiac.2016.02.012 27066882

[B37] WHO (2017). *Global Priority List of Antibiotic-Resistant Bacteria to Guide Research, Discovery, and Development of New Antibiotics*. Available at: http://www.who.int/medicines/publications/WHO-PPL-Short_Summary_25Feb-ET_NM_WHO.pdf

[B38] Wilke M. S. Hills T. L. Zhang H. Z. Chambers H. F. Strynadka N. C. (2004). Crystal structures of the Apo and penicillin-acylated forms of the BlaR1 beta-lactam sensor of *Staphylococcus aureus*. *J. Biol. Chem.* 279 47278–47287. 10.1074/jbc.M407054200 15322076

[B39] Wilson D. N. Schluenzen F. Harms J. M. Starosta A. L. Connell S. R. Fucini P. (2008). The oxazolidinone antibiotics perturb the ribosomal peptidyl-transferase center and effect tRNA positioning. *Proc. Natl. Acad. Sci. U.S.A.* 105 13339–13344. 10.1073/pnas.0804276105 18757750 PMC2533191

[B40] Wiltshire M. D. Foster S. J. (2001). Identification and analysis of *Staphylococcus aureus* components expressed by a model system of growth in serum. *Infect. Immun.* 69 5198–5202. 10.1128/IAI.69.8.5198-5202.2001 11447207 PMC98621

[B41] Woods C. Colice G. (2014). Methicillin-resistant *Staphylococcus aureus* pneumonia in adults. *Expert Rev. Respir. Med.* 8 641–651. 10.1586/17476348.2014.940323 25030040

[B42] Zhang Y. Luo M. Zu Y. Fu Y. Gu C. Wang W. (2012). Dryofragin, a phloroglucinol derivative, induces apoptosis in human breast cancer MCF-7 cells through ROS-mediated mitochondrial pathway. *Chem. Biol. Interact.* 199 129–136. 10.1016/j.cbi.2012.06.007 22796323

[B43] Zhao D. D. Zhao Q. S. Liu L. Chen Z. Q. Zeng W. M. Lei H. (2014). Compounds from *Dryopteris fragrans* (L.) Schott with cytotoxic activity. *Molecules* 19 3345–3355. 10.3390/molecules19033345 24647035 PMC6271107

[B44] Zhao J. Cheah S.-E. Roberts K. D. Nation R. L. Thompson P. E. Velkov T. (2016). Transcriptomic analysis of the activity of a novel polymyxin against *Staphylococcus aureus*. *mSphere* 1:e00119-16. 10.1128/mSphere.00119-16 27471750 PMC4963539

[B45] Zhong Z.-C. Zhao D.-D. Liu Z.-D. Jiang S. Zhang Y.-L. (2017). A new human cancer cell proliferation inhibition sesquiterpene, dryofraterpene a, from medicinal plant *Dryopteris fragrans* (L.) Schott. *Molecules* 22:180. 10.3390/molecules22010180 28117728 PMC6155874

[B46] Zou L. Wang J. Huang B. Xie M. Li A. (2010). A solute-binding protein for iron transport in *Streptococcus iniae*. *BMC Microbiol.* 10:309. 10.1186/1471-2180-10-309 21122131 PMC3014919

