# Supplementary figures and images for: Antibacterial Activity and Mechanism of Action of Aspidinol Against Multi-Drug-Resistant Methicillin-Resistant Staphylococcus aureus
Source: Front Pharmacol. 2018 Jun 13;9:619. doi: 10.3389/fphar.2018.00619 (PMC6008372; doi:10.3389/fphar.2018.00619)

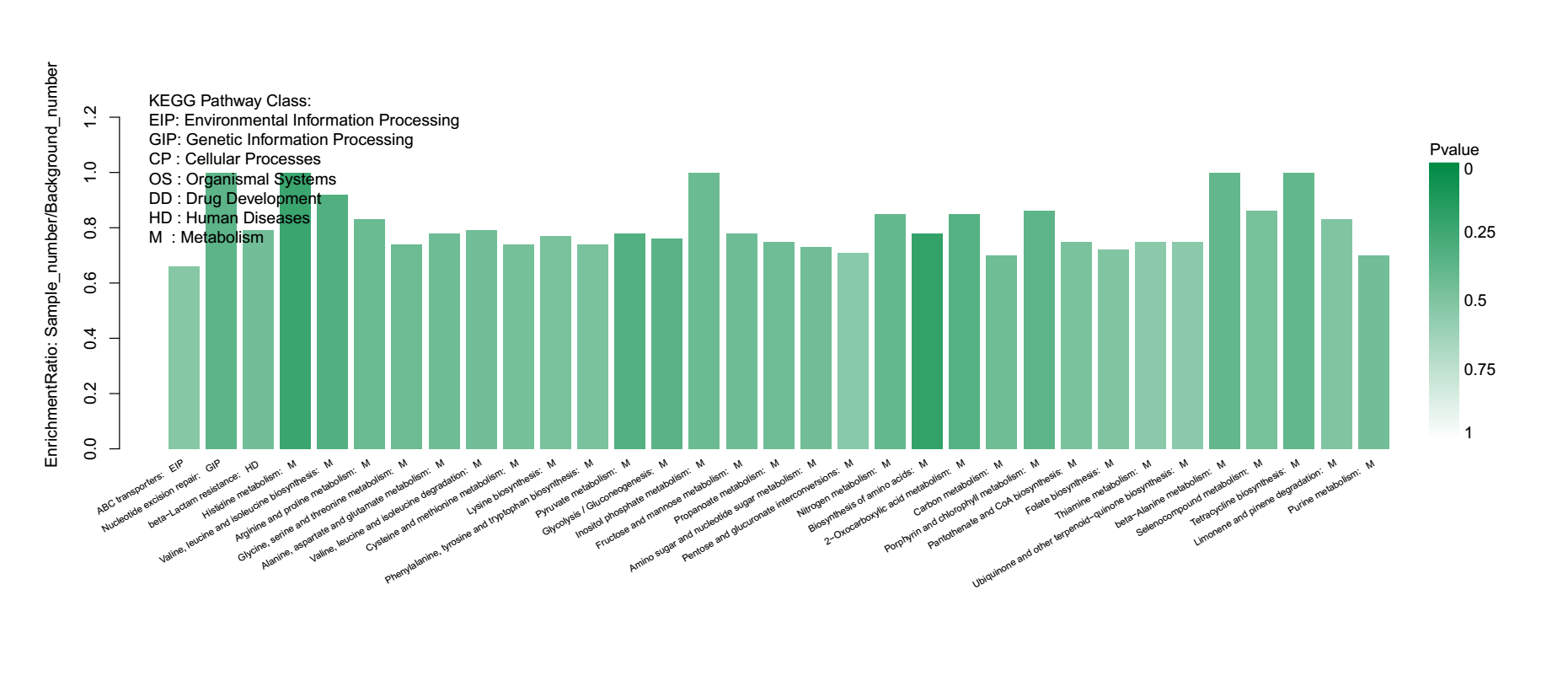

Supplement: FIGURE S1 — Differentially expressed genes enriched in the KEGG pathway for S. aureus ATCC 33591 cells treated and not treated with aspidinol. [file Image_1.TIF]

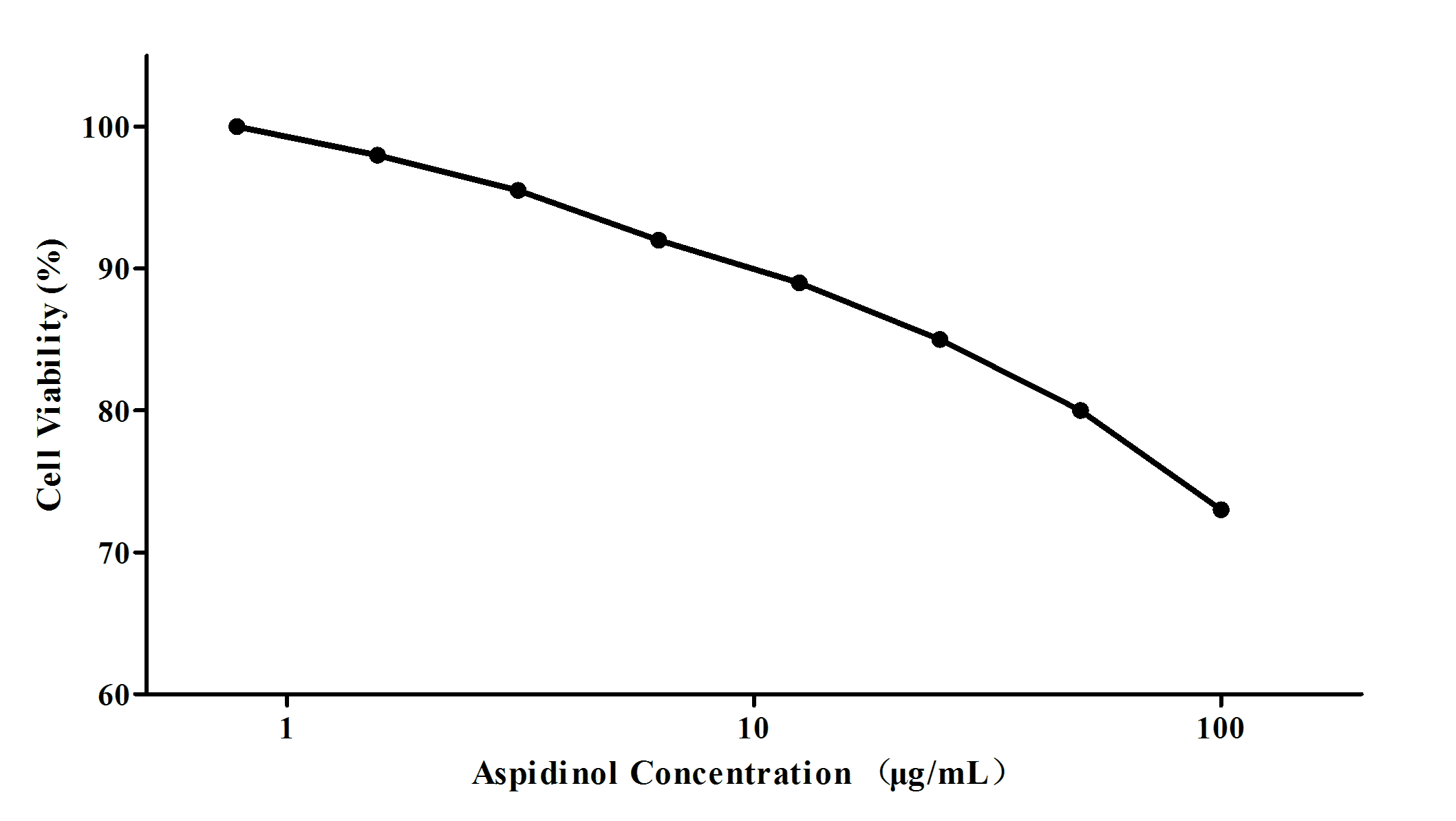

Supplement: FIGURE S2 — Cell survival curve after treated with aspidinol. [file Image_2.TIF]

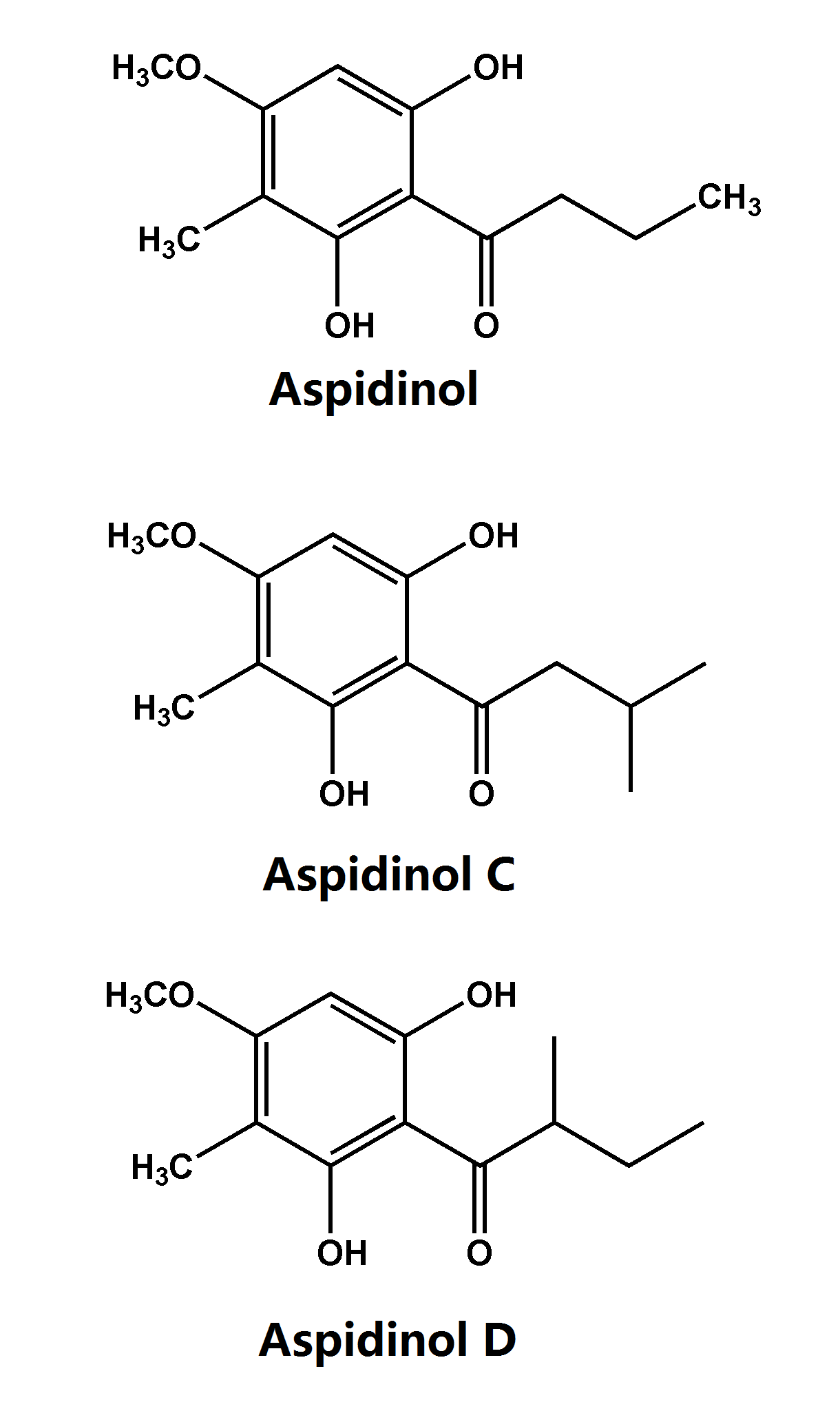

Supplement: FIGURE S3 — Chemical structure of aspidinol, aspidinol C and aspidinol D. [file Image_3.TIF]
