# Supplementary material for: Antibacterial Activity and Mechanism of Action of Aspidinol Against Multi-Drug-Resistant Methicillin-Resistant Staphylococcus aureus
Source: Front Pharmacol. 2018 Jun 13;9:619. doi: 10.3389/fphar.2018.00619 (PMC6008372; doi:10.3389/fphar.2018.00619)
Supplement: TABLE S1 — Strain list. [file Table_1.DOCX]

**Table S1.** Strain list

| Strains | Description | Reference |
| --- | --- | --- |
|  |  |  |
| MSSA |  |  |
| ATCC29213 | MSSA;Sensitive to oxacillin | 1 |
| L1 | MSSA;human throat swab isolate;2010 | This study |
| L2 | MSSA;human throat swab isolate;2010 | This study |
| L4 | MSSA;human throat swab isolate;2010 | This study |
| L5 | MSSA;human throat swab isolate;2010 | This study |
| L7 | MSSA;human throat swab isolate;2010 | This study |
| L11 | MSSA;human throat swab isolate;2010 | This study |
| L13 | MSSA;human throat swab isolate;2010 | This study |
| L17 | MSSA;human throat swab isolate;2010 | This study |
| L22 | MSSA;human throat swab isolate;2010 | This study |
| L23 | MSSA;human throat swab isolate;2010 | This study |
| L24 | MSSA;human throat swab isolate;2010 | This study |
| L28 | MSSA;human throat swab isolate;2010 | This study |
| L30 | MSSA;human throat swab isolate;2010 | This study |
| L31 | MSSA;human throat swab isolate;2010 | This study |
| L32 | MSSA;human phlegm isolate;2010 | This study |
| L37 | MSSA;human phlegm isolate;2010 | This study |
| L40 | MSSA;human phlegm isolate;2010 | This study |
| L55 | MSSA;human phlegm isolate;2010 | This study |
| L56 | MSSA;human phlegm isolate;2010 | This study |
| L57 | MSSA;human phlegm isolate;2010 | This study |
|  |  |  |
| MRSA |  |  |
| ATCC33591 | MRSA;SCCmec: Type III;spa type Ridom: t037;spa type Kreiswirth: WGKAOMQ; pvl gene amplification: Negative | 2 |
| LN2 | MRSA;human throat swab isolate;2010 | This study |
| LN3 | MRSA;human throat swab isolate;2010 | This study |
| LN4 | MRSA;human throat swab isolate;2010 | This study |
| LN6 | MRSA;human throat swab isolate;2010 | This study |
| LN8 | MRSA;human throat swab isolate;2010 | This study |
| LN18 | MRSA;human phlegm isolate;2010 | This study |
| LN19 | MRSA;human phlegm isolate;2010 | This study |
| LN20 | MRSA;human phlegm isolate;2010 | This study |
| LN21 | MRSA;human phlegm isolate;2010 | This study |
| LN22 | MRSA;human phlegm isolate;2010 | This study |
| LN23 | MRSA;human phlegm isolate;2010 | This study |
| LN30 | MRSA;human phlegm isolate;2010 | This study |
| LN33 | MRSA;human phlegm isolate;2010 | This study |
| LN36 | MRSA;human phlegm isolate;2010 | This study |
| LN44 | MRSA;human phlegm isolate;2010 | This study |
| LN45 | MRSA;human phlegm isolate;2010 | This study |
| LN46 | MRSA;human phlegm isolate;2010 | This study |
| LN50 | MRSA;human phlegm isolate;2010 | This study |
| LN58 | MRSA;human urine isolate;2010 | This study |
| LN63 | MRSA;human urine isolate;2010 | This study |

1. Performance Standards for Antimicrobial Disk Susceptibility Tests; Approved Standard - 9th Edition. Wayne, PA. Clinical and Laboratory Standards Institute; CLSI M2-A9.

2. Schaefler S, et al. Methicillin-resistant strains of Staphylococcus aureus phage type 92. Antimicrob. Agents Chemother. 15: 74-80, 1979. PubMed: [154874](http://www.ncbi.nlm.nih.gov/sites/entrez?cmd=Retrieve&db=PubMed&list_uids=154874&dopt=AbstractPlus).
