# Supplementary material for: Antibacterial Activity and Mechanism of Action of Aspidinol Against Multi-Drug-Resistant Methicillin-Resistant Staphylococcus aureus
Source: Front Pharmacol. 2018 Jun 13;9:619. doi: 10.3389/fphar.2018.00619 (PMC6008372; doi:10.3389/fphar.2018.00619)
Supplement: TABLE S2 — Primers used in this study. [file Table_2.DOCX]

**Table S2.** Primers used in this study.

| Gene | Primer direction | Primer sequence (5’→3’) |
| --- | --- | --- |
| *ilvB* | Sense | GGCTGAATCACTTGAACTT |
|  | Antisense | ACCAGGATAACCGAATAAA |
| *ilvC* | Sense | TGACTATGTTTCAGGACCAC |
|  | Antisense | GTAATTCACGACCAACTTTT |
| *ilvD* | Sense | AGCACCAGCAAGAAGT |
|  | Antisense | GTCCAGGAACAATATCAA |
| *lysC* | Sense | ACGCATCCATCGTTCATA |
|  | Antisense | CCACCAACTGTGCCTAAT |
| *patA* | Sense | CCGATGCCTGATGTTGT |
|  | Antisense | TTGCTTCACTTGCTCCA |
| *oppA* | Sense | GACTTAACATCCCTTGA |
|  | Antisense | ACCATTGCTCCATTT |
| *oppB* | Sense | GGATTTGATTAAACCGAGAC |
|  | Antisense | AGCTGCTGCAACACCTA |
| *oppC* | Sense | TTAGGTGCTATCGTGGTT |
|  | Antisense | TGTTTGAGGTGCAGGTAC |
| *oppD* | Sense | ATTTGCGAGACGTAGTCA |
|  | Antisense | ATTAGGCATCGGTTTCAT |
| *oppF* | Sense | TTATCAGCCATTCCACAA |
|  | Antisense | GCCGCTTCTTCTTCAGTA |
| *mtsA* | Sense | AAAATGTTGGTGGAGA |
|  | Antisense | TAACGTCAGCGTCAGT |
| *mtsB* | Sense | CGAGCATTGATAACTT |
|  | Antisense | GATAGGGCAACACCAG |
| *mtsC* | Sense | CGAATGGTGCTGGTAA |
|  | Antisense | TGTTGCAGTTGTTGTTGTA |
| *fhuA* | Sense | TACGGGGATAACACGA |
|  | Antisense | TAACTGCCAATAAACG |
| *fhuB* | Sense | AGCAATCAACAGCAAAAC |
|  | Antisense | CAGCAAGACCATTACGAG |
| *fhuG* | Sense | AAGACGCACAACACTC |
|  | Antisense | AATCATCCTTGGCATA |
| *isdA* | Sense | ATTACAATGGGTACAGC |
|  | Antisense | TATTAGTTGCGTTCGT |
| *estA* | Sense | CCGAAACAGTTAGAAA |
|  | Antisense | AGACGGCAATGAGAAT |
| *RP-S20* | Sense | ATGGCAAATATCAAATC |
|  | Antisense | CTTTTGTGAAATGTTGC |
| *RP-L20* | Sense | AGGTGGAACAGTAACAA |
|  | Antisense | GTCACGGAAAGCATAT |
| *RP_L1* | Sense | AAGAAGCAGCTAGTAAA |
|  | Antisense | CAACAGAAGCGTCAAA |
| *grpE* | Sense | AAACATATCAAGCACAACG |
|  | Antisense | CAAACTTTCATGCACCATT |
| *sav1327* | Sense | AAGATCGCATCCCTGTGG |
|  | Antisense | AGCCGACTTGTCTATTCATT |
| *sav1852* | Sense | GCGCAAACATTCATTA |
|  | Antisense | ATTTAGAGCAACAAGC |
